# The role of rumination and racing thoughts in co-occurrence of ADHD symptoms, depression and anxiety symptoms

**DOI:** 10.1080/00049530.2026.2679669

**Published:** 2026-06-01

**Authors:** Bethany Devenish, Luisa Weiner, Nicole Papadopoulos

**Affiliations:** a Faculty of Education, Monash University, Melbourne, Victoria, Australia; b Department of Psychology, Université de Strasbourg, Strasbourg, France; c Health and Social Care Unit, School of Public Health and Preventive Medicine, Monash University, Melbourne, Victoria, Australia

**Keywords:** Racing thoughts, rumination, ADHD, depression, anxiety

## Abstract

**Objective:**

Higher rates of comorbidity for depressive disorders and anxiety disorders have been observed in adults with Attention-Deficit/Hyperactivity Disorder (ADHD), which result in a significantly poorer prognosis. Developing an understanding of why this comorbidity occurs may help identify modifiable factors and inform treatment approaches. However, there is limited research examining the mechanisms through which these symptoms co-occur. This study evaluated whether racing thoughts and rumination (which have been observed in adults with ADHD, depression and anxiety) mediate the relationship between inattentive and hyperactive/impulsive symptoms of ADHD and depression and anxiety symptoms in a general population sample.

**Method:**

Participants were 289 adults, aged 18–65 (*M* = 38.57; SD = 11.24; 64% female), who completed online self-report questionnaires assessing their symptoms of inattention and hyperactivity/impulsivity, racing thoughts, rumination, and depression and anxiety symptoms.

**Results:**

Racing thoughts significantly mediated the relationship between inattentive and hyperactive/impulsive symptoms of ADHD with depression and anxiety symptoms. Rumination significantly mediated the relationship between hyperactive/impulsive symptoms, but not inattentive symptoms, with depression and anxiety symptoms.

**Conclusion:**

Implications for future research and interventions are outlined in relation to the role racing thoughts and rumination may play in the association between ADHD symptoms and depression and anxiety symptoms.

## KEY POINTS

**What is already known about this topic:**

1. There is a lack of clarity as to why there is high comorbidity between attention deficit hyperactivity disorder (ADHD), depressive disorders, and anxiety disorders.
2. Rumination is a key target for interventions for depression and anxiety, with some evidence suggesting rumination may mediate the relationship between ADHD and depression and anxiety.
3. Racing thoughts has been recently identified as a potential transdiagnostic factor due to its increased presence in people with ADHD, depression or anxiety.

**What this study adds:**

1. This study indicated that racing thoughts may play a significant role in the relationship between inattentive and hyperactive/impulsive symptoms with depression and anxiety symptoms in a general population sample.
2. In comparison, despite being a key target for many depression and anxiety interventions, rumination appeared to only be a significant contributor to the relationship between hyperactive/impulsive symptoms with depression and anxiety symptoms.
3. While further research with clinical populations is needed, these findings highlight the importance of exploring the effectiveness of treatment approaches that target racing thoughts in people with increased ADHD, anxiety or depression.

Attention-deficit/hyperactivity disorder (ADHD) is a neurodevelopmental condition that involves consistent differences in attention, hyperactivity and impulse control which negatively impact a person’s functioning (American Psychiatric Association, 2022). There are three presentations of ADHD, specifically predominantly inattentive, predominantly hyperactive-impulsive, or combined, depending on whether an individual has met enough criteria for symptoms of inattention, hyperactivity-impulsivity, or both, respectively (APA, 2022). Increased comorbidity with mental health disorders has been observed in adults with ADHD (Choi et al., 2022), which results in greater functional severity and impairment, and increased suicidality, than when these conditions present alone (DiLorenzo et al., 2021). As such, developing an understanding of the mechanisms through which these symptoms co-occur may help identify modifiable factors, and inform treatment approaches.

There is a lack of clarity as to the explanatory relationship between ADHD and co-occurring depression or anxiety. Symptoms of ADHD may interact with or increase depression and anxiety symptoms, due to shared mechanisms (e.g., rumination tendency) and indirect pathways (e.g., experiencing social exclusion related to symptoms; D’Agati et al., 2019). Additionally, these conditions may also coexist due to overlapping genetic (Faraone & Larsson, 2019) and neuropsychological factors (Snyder et al., 2015). For example, meta-analyses on adults with depression, anxiety or ADHD symptoms have identified altered connectivity within and between the default mode network (DMN, i.e., a network thought to be active during internal reflection, including mind wandering and daydreaming states; Sambucco et al., 2022; Sutcubasi et al., 2020), cognitive control network, and affective control network (Doucet et al., 2020; Kolesar et al., 2019; Lukito et al., 2020; Sutcubasi et al., 2020; Wang et al., 2022), suggesting that related difficulties could underlie the comorbidity between ADHD, depression and anxiety. In particular, two cognitive processes – i.e., rumination and racing thoughts – have been linked both to mind wandering (Kandeğer et al., 2023; Martz, Weiner, Bonnefond, et al., 2023) and depression, as well as anxiety symptoms, in people with ADHD (Bertschy et al., 2020; Jonkman et al., 2017; Kandeğer et al., 2023; Nolen-Hoeksema, 2000; Yeguez et al., 2018). Hence, this study focuses on rumination and racing thoughts as potential mediators of the relationship between ADHD symptoms and depression and anxiety symptoms.

## Ruminative thinking style

Ruminative thinking styles are a form of internal reflection that consist of a stable response pattern to discrepancies between a person’s current and desired state, involving excessive, persevering, and passive thinking about negative feelings and distress and their associated causes and consequences, that is difficult to control or stop (Nolen-Hoeksema et al., 2008; Smith & Alloy, 2009). Rumination is often viewed dichotomously as reflective rumination (i.e., actively thinking about solutions) and brooding rumination (i.e., passively focusing on distress; Treynor et al., 2003). Rumination is a key target for interventions for depression and anxiety (Querstret & Cropley, 2013), yet little literature has explored the possibility that rumination may be a mediating pathway between symptoms of ADHD, and depression and anxiety symptoms. A recent study on adults with ADHD identified rumination as a mediating factor for the relationship between ADHD symptoms and a composite measure of depression and anxiety (Kandeğer et al., 2023). Similarly, another study identified brooding rumination, but not reflective rumination, as a mediator of the relationship between inattentive and hyperactivity/impulsivity symptoms, and depression symptoms, even when accounting for an additional indirect pathway through interpersonal conflict (Horibe & Hasegawa, 2020).

In contrast to findings indicating that rumination may mediate the relationship between ADHD and depression and anxiety symptoms, a study by Fredrick et al. (2020) found that ADHD symptoms were no longer significantly associated with rumination when cognitive disengagement syndrome (CDS; also known as sluggish cognitive tempo) was included in a regression model. CDS involves slower paced behaviour and thinking, excessive daydreaming, and mental confusion and fogginess, and is increasingly being recognised as being clinically distinct from ADHD presentations (Burns et al., 2025). This finding is of interest, as current models related to depression and anxiety have focused on the ways attentional and executive functioning difficulties may disrupt an individual’s ability to redirect attention away from negative thoughts (see for example, Beevers, 2005; Eysenck et al., 2007), but little attention has been paid to the role an individual’s pace of thinking may play. CDS has been linked to differences in the DMN (Camprodon-Rosanas et al., 2019), executive functioning (Camprodon-Rosanas et al., 2019) and attentional control (Camprodon-Rosanas et al., 2019; Kardaş et al., 2021) regions of the brain, with a number of studies indicating CDS is associated with symptoms of ADHD, anxiety and depression (Becker et al., 2013, 2018; Fredrick et al., 2021; Kamradt et al., 2022). Given that CDS seems to be linked to depression, anxiety and ADHD symptoms (Becker et al., 2013, 2018; Fredrick et al., 2021; Kamradt et al., 2022), and to regions of the brain associated with attention and internal reflection (Camprodon-Rosanas et al., 2019; Kardaş et al., 2021), and that CDS appears to account for the relationship between ADHD symptoms and rumination (Fredrick et al., 2020), it is possible that the pace of one’s thinking could be a stronger explanatory factor for the relationship between ADHD, depression and anxiety than ruminative thinking.

## Racing thoughts

**Figure 1.**
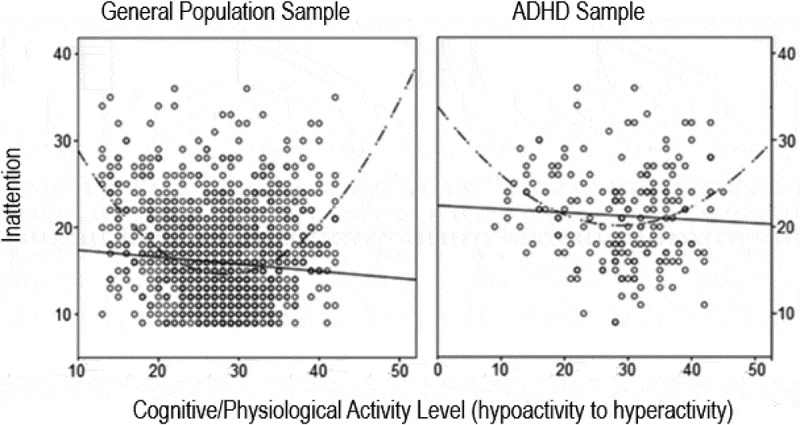
U-shape relationship between cognitive/physiological activity and inattention.

*Note*. Adapted with permission from Miller & Prevatt (2020).

A growing number of studies have examined the interrelationships between internal thinking styles, depression, anxiety, and ADHD symptoms in relation to CDS, however few studies have considered the potential inverse cognitive pace of thinking – that is, whether a quickened cognitive tempo akin to cognitive hyperactivity may also interact with symptoms of ADHD, depression, and anxiety. While early studies suggested that CDS may help identify a predominantly inattentive presentation of ADHD (Bauermeister et al., 2005; Milich et al., 2001), following studies were unable to substantiate this, instead finding CDS presents across all presentations of ADHD (Becker, 2021; Becker et al., 2016). Subsequent research suggested that attention may have an inverted U-shape relationship with a continuum of cognitive activity, as depicted in Figure 1, with increased symptoms of inattention presenting at higher levels of hypoactivity (e.g., CDS) or hyperactivity (Miller & Prevatt, 2020).

One form of cognitive hyperactivity that may be particularly relevant for adults with ADHD are racing thoughts. Racing thoughts are a distinct construct to rumination (Bertschy et al., 2020), and involve an overproduction of thoughts that are characterised by acceleration in the speed at which they occur (Piguet et al., 2010). Research over several decades has identified racing thoughts in mood disorders (Benazzi, 2003; Bertschy et al., 2020; Braden & Ho, 1981; Piguet et al., 2010), with more recent research suggesting racing thoughts may also be linked to anxiety symptoms (Bertschy et al., 2020; Weiner et al., 2021) and ADHD (Bertschy et al., 2020; Martz et al., 2021). Consequently, it has been suggested that racing thoughts may be a potential transdiagnostic thinking style due to its strong links to affective dysregulation and hyperarousal in both clinical and general populations (Weiner et al., 2021).

Martz et al. (2021) suggest that racing thoughts may be a form of “thought hyperactivity” experienced by people with ADHD, with research in ADHD populations finding racing thoughts were linked to emotional lability (Martz et al., 2021; Martz, Weiner, Bonnefond, et al., 2023), motor hyperactivity (Martz et al., 2021), hyperarousal (Martz et al., 2021) and other ADHD symptomology (e.g., risk-taking; Martz, Weiner, Bonnefond, et al., 2023), and were predictive of interpersonal problems in people with ADHD (Martz, Weiner, Bonnefond, et al., 2023). Further, adults with combined presentations of ADHD appear to experience increased racing thoughts compared to adults with inattentive presentations only (Bertschy et al., 2023; Martz et al., 2021), with one study finding that in a subsample of adults with an inattentive presentation of ADHD, racing thoughts were not significantly related to inattentive symptoms of ADHD when variance associated with hyperactivity symptoms and anxiety symptoms was accounted for (Martz et al., 2021).

Racing thoughts are theorised to occur due to reduced cognitive control (Piguet et al., 2010), and/or overactivation of the semantic network (Bertschy et al., 2023; Martz et al., 2022), with both theories implicating DMN and executive functioning networks in the brain that are also associated with depression and anxiety symptoms (Backes et al., 2014; Hu et al., 2021; Ronold et al., 2020; Takamura et al., 2017). As such, while there is limited research examining racing thoughts in adults with ADHD symptomatology, it is plausible that racing thoughts may also be a contributing factor to the relationship between ADHD, depression and anxiety symptoms.

## The current study

**Figure 2.**
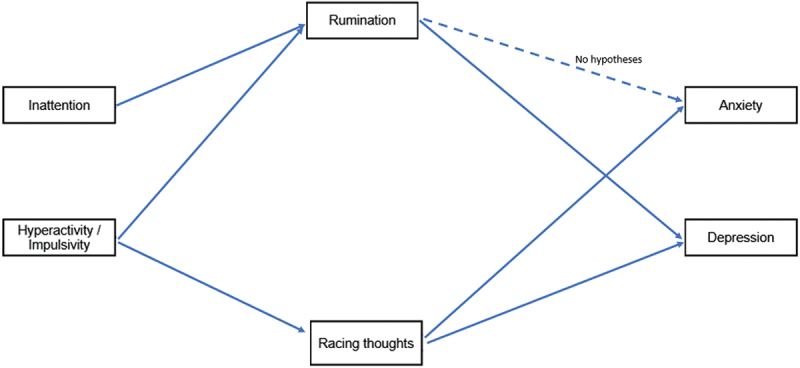
Hypothesised pathways in structural equation model.

Many interventions for adults experiencing depression or anxiety disorders target rumination (Querstret & Cropley, 2013), however there appears to be little research examining whether rumination is indeed a strong contributor to comorbid depression or anxiety symptoms in adults with ADHD symptoms. Further, little research has explored the possibility that racing thoughts may be a contributor to the comorbidity between ADHD and depression and anxiety. Specifically, to our knowledge, there appears to be no research examining the extent to which rumination and racing thoughts may together explain the relationship between ADHD symptoms and depression and anxiety symptoms. Developing an understanding of the pathways through which higher levels of inattention or hyperactivity/impulsivity may co-present with mood or anxiety symptoms in adults appears critical for informing effective treatment approaches. Symptoms of ADHD are known to exist across the general population (Vogel et al., 2018), and subclinical or even low levels of ADHD symptoms can increase rumination (Horibe & Hasegawa, 2020), depression symptoms (Horibe & Hasegawa, 2020; Vogel et al., 2018), and anxiety symptoms (Vogel et al., 2018). The current study therefore aims to examine whether rumination and racing thoughts mediate the relationship between ADHD symptoms and depression and anxiety symptoms in a general population sample. The study research questions and hypotheses are outlined below. Figure 2 provides a visual summary of the hypothesised model.

Does rumination mediate the relationship between inattentive and hyperactive/impulsive symptoms of ADHD and depression?
  **Hypothesis One**: Given previous research has identified rumination mediates the relationship between inattentive symptoms of ADHD and depression (Horibe & Hasegawa, 2020; Kandeğer et al., 2023), it was predicted that rumination would be a significant pathway between these variables.
  **Hypothesis Two**: Given previous research has identified rumination mediates the relationship between hyperactive/impulsive symptoms of ADHD and depression (Horibe & Hasegawa, 2020; Kandeğer et al., 2023), it was predicted that rumination would be a significant pathway between these variables.
2. Does rumination mediate the relationship between inattentive and hyperactive/impulsive symptoms of ADHD and anxiety?
  No hypothesis was generated for the pathways between inattentive and hyperactive/impulsive symptoms of ADHD, rumination and anxiety symptoms due to limited and conflicting findings
  (Fredrick et al., 2020; Kandeğer et al., 2023).
Do racing thoughts mediate the relationship between inattentive and hyperactive/impulsive symptoms of ADHD and depression?
  **Hypothesis Three**: Given the limited previous research on the relationship between inattentive symptoms and racing thoughts suggests this relationship may not exist once hyperactivity symptoms and anxiety symptoms are accounted for (Martz et al., 2021), it was predicted that no significant pathways to depression would be identified from inattentive symptoms to racing thoughts.
  **Hypothesis Four**: Given previous research has identified racing thoughts are associated with hyperactive symptoms of ADHD (Bertschy et al., 2020; Martz et al., 2021) and mood symptoms (Benazzi, 2003; Bertschy et al., 2020; Braden & Ho, 1981; Piguet et al., 2010), it was predicted that racing thoughts would be a significant pathway between hyperactive/impulsive symptoms and depression.
Do racing thoughts mediate the relationship between inattentive and hyperactive/impulsive symptoms of ADHD and anxiety?
  **Hypothesis Five**: Given the limited previous research on the relationship between inattentive symptoms and racing thoughts suggests this relationship may not exist once hyperactivity symptoms and anxiety symptoms are accounted for (Martz et al., 2021), it was predicted that no significant pathways to anxiety would be identified from inattentive symptoms to racing thoughts.
  **Hypothesis Six**: Given previous research has identified racing thoughts are associated with hyperactive symptoms of ADHD (Bertschy et al., 2020; Martz et al., 2021) and with anxiety (Bertschy et al., 2020; Weiner et al., 2021) it was predicted that racing thoughts would be a significant pathway between hyperactive/impulsive symptoms and anxiety.

## Materials and methods

### Participants and procedures

In September and October 2022, adults aged 18 to 65 years were invited to view the study details, and complete an online survey. To be included in this study, participants needed to be aged 18 to 65 years. Recruitment occurred via online posts in Australian university, psychology, and research Facebook group pages (with permission from group administrators) and via CloudResearch (a global survey recruitment platform). The survey was administered in English, and therefore CloudResearch recruitment primarily targeted adults living in the United States of America. A total of 289 adults (64% female; *M* age = 38.57 years, *SD* = 11.24) who provided informed consent online and completed the 20-minute survey were included in the study. Participants recruited via CloudResearch (196 participants) received a payment of $2 for their participation, and participants recruited via social media (93 participants) could enter an anonymous draw to win one of four $25 gift vouchers.

The demographic details of study participants are provided in Table 1. Respondents on the CloudResearch platform whose responses were indicated as poor quality, defined as response times of less than one item per second (Wood et al., 2017), “unusual responses” to open-ended questions (Chmielewski & Kucker, 2020), or consistently low standard deviation of responses to scales (Collier, 2020), were excluded from the study (*n* = 31). No other exclusion criteria applied. Ethical approval was provided by the Monash University Human Research Ethics Committee (Project ID: 34,716).

**Table 1.** Participant characteristics.

Characteristic	Values *N* (%)
Country of birth.	
Australia	74 (26%)
US	178 (63%)
Other (Afghanistan; Brazil; Canada; China; Croatia; Fiji; Germany; India; Japan; Malaysia; Morocco; New Zealand; Philippines; Portugal; Romania; South Africa; South Korea; Taiwan; United Kingdom)	28 (10%)
Highest Level of Education.	
Postgraduate	37 (13%)
Undergraduate	72 (25%)
Vocational Certificate or Diploma	96 (34%)
Secondary	63 (28%)
Did not complete secondary schooling	16 (6%)
Employment Status.	
Casual	9 (3%)
Part-time	40 (14%)
Full-time	131 (46%)
Self-employed	23 (8%)
Student	9 (3%)
Unemployed	31 (11%)
Caring for family or others in unpaid capacity	21 (7%)
Retired	20 (7%)
Taking medication that may impact attention	41 (14%)
Taking medication that may impact mood	67 (24%)

### Measures

Demographic variables included age, gender, country of birth, education, employment, and two dichotomous measures indicating presence or absence of medication that may impact attention or mood.

#### Inattention and hyperactivity/impulsivity characteristics

The five-point, nine-item inattentive and hyperactivity/impulsivity subscales of the Adult ADHD Self-Report Scale Symptom Checklist (ASRS-v1.1; Kessler et al., 2005) were administered. The inattentive subscale assesses difficulties over the past six months in concentration, focusing on detail, and organisation (total range = 0 - 36), and the hyperactivity/impulsivity subscale assesses difficulties over the past six months in sitting still or relaxing, and inhibiting talking and interrupting others (total range = 0 - 36). Scores of ≥ 6 on either subscale are considered symptomatic (Adler et al., 2019), with high sensitivity and moderate specificity (Hines et al., 2012).

Evaluations of the ASRS have identified good reliability and validity (Adler et al., 2006; Brevik et al., 2020; van de Glind et al., 2013). Using Cicchetti’s (1994) criteria for reliability, Cronbach’s alpha indicated high reliability in our study sample for the total scale (Cronbach’s α = .94), inattentive subscale (Cronbach’s α = .91), and hyperactivity/impulsivity subscale (Cronbach’s α = .88). A CFA was conducted to confirm the measurement model. In keeping with recommendations, the relative chi-square of 2.59 was greater than 1.0 (Byrne, 1989) and less than 3.0 (Kline, 2015). Similarly, CFI, TLI and IFI indices were all > .90 (.92, .91, and .92 respectively), and RMSEA was between .05 and .08, indicating acceptable fit was achieved (Hu & Bentler, 1999; MacCallum et al., 1996). All standardised regression weights were significant (below the .001 level), and all items were greater than > .50 (range = .57 - .74).

#### Rumination

Rumination was measured using the Ruminative Response Scale (RRS-10; Treynor et al., 2003). The RRS-10 consists of ten items that assess the frequency of ruminative thoughts and behaviours from 1 (almost never) to 4 (almost always).

Cronbach’s alpha indicated good reliability for the total scale, brooding subscale and reflection subscale (Cronbach’s α = .89, .82, and .81 respectively). The measurement model fit for the two-factor model was mediocre in the current study, which is in keeping with a recent study that suggested the scale may be better constructed as a unidimensional scale (Valencia & Paredes-Angeles, 2022). The measurement model for a unidimensional scale was therefore assessed. In keeping with previous studies (Parola et al., 2017; Valencia & Paredes-Angeles, 2022), CFA indicated low factor loading items for one item ‘’write down what you are thinking and analyse it”. This item was therefore excluded from the current study. Following removal of this item, in keeping with recommendations, the relative chi-square of 2.54, CFI, TLI and IFI indices of .97, .97, and .96 respectively, and RMSEA of .07 indicated acceptable fit was achieved. All standardised regression weights were significant (below the .001 level), all items were greater than > .50 (range = .61 - .80), and Average Variance Extracted was .69. Given measurement fit for the unidimensional model was within expected limits, and correlation analyses indicated no inconsistencies between the original subscale items with outcome variables, the unidimensional model of rumination was used for all analyses.

#### Racing thoughts

Racing thoughts were measured using the Racing and Crowded Thoughts Questionnaire (RCTQ-13; Weiner et al., 2019). The RCTQ-13 consists of 13 items that assess racing thoughts across three factors including (1) thought activation, (2) burden of thought activation, and (3) thought overexcitability. In the present study, a 4-point scale was administered, ranging from not at all (1), to completely agree (4). Items were summed (total range = 13 - 52), with higher scores indicating increased presence of racing thoughts. Evaluations of the RCTQ-13 have identified good reliability and reliability in clinical populations (Weiner et al., 2019). Cronbach’s alpha identified good reliability for the total scale, and for the thought activation, burden of thought activation, and thought overexcitability subscales (Cronbach’s α = .95, .89, .90, and .88 respectively). A CFA was conducted to confirm the scale validity was maintained when using a 4-point response scale. All standardised regression weights were significant (below the .001 level), and all items were greater than > .60 (range = .66 - .88), which indicates all items explained an acceptable amount of the variance for each subscale (Hair et al., 2017; Kline, 2015). In keeping with recommendations, the relative chi-square of 2.54 was greater than 1.0 (Byrne, 1989) and less than 3.0 (Kline, 2015). Similarly, CFI, TLI and IFI indices were all > .90 (.97, .96, and .97 respectively), and RMSEA was between .05 and .08, indicating acceptable fit was achieved (Hu & Bentler, 1999; MacCallum et al., 1996). Average Variance Extracted for each subscale was > .50 (overactivated = .69, excited = .61, and burden = .70), indicating good convergent validity (Kang & Ahn, 2021). In sum, the CFA indicates the validity of subscales was maintained.

#### Depression and anxiety symptoms

The 7-item anxiety and depression subscales of the Depression Anxiety Stress Scale (DASS-21; Lovibond & Lovibond, 1995) were administered. Response items are measured on a 4-point scale ranging from “did not apply to me at all” (0) to “applied to me very much or most of the time” (3). The depression subscale assesses symptoms of depression such as dysphoria, inertia and hopelessness (total range = 0 - 42), and the anxiety subscale assesses physiological (e.g., trembling) and subjective experiences of anxiety (e.g., feeling scared without a good reason). Items are summed and multiplied by 2 to create a range of 0–126, with higher scores indicating higher psychological distress.

Evaluations of the DASS-21 have identified good reliability and validity in clinical and non-clinical populations in westernised countries (Antony et al., 1998; Henry & Crawford, 2005; Lee, 2019). Cronbach’s alpha indicated high reliability in our study sample for the total scale (Cronbach’s α = .96), anxiety subscale (Cronbach’s α = .90), and depression subscale (Cronbach’s α = .93). Confirmatory factor analysis conducted in AMOS supported the validity of the measurement model in our study sample. Specifically, the relative chi-square of 2.40, CFI, TLI and IFI indices of .96, .96, and .96 respectively, and RMSEA of .07 indicated acceptable fit was achieved. Further, all standardised regression weights were significant (below the .001 level), and all items were greater than > .50 (range = .58 - .85).

### Statistical analysis

Assumption testing, demographic characteristics and scale reliability analyses were conducted using IBM SPSS Statistics version 29. Structural equation modelling of a parallel mediation model using maximum likelihood estimation was conducted in IBM SPSS AMOS version 29. Covariances were included for ASRS and DASS subscales. Residual covariances between all mediators were estimated.

Data were checked for reliability (i.e., internally inconsistent responses on validated scales; Peer et al., 2022) and responses to open-ended questions that bordered on “unusual” (Chmielewski & Kucker, 2020). Two of these responses were identified as multivariate outliers by Mahalanobis distance and Cook’s distance, and were therefore excluded from analyses. A further 3 participants were excluded due to missing data, leaving a final sample of 284 participants.

An a priori estimate of sample size based on the RMSEA statistic was conducted using Preacher and Coffman’s (2006) online tool. Calculations for three sets of *df*s were conducted (50, 80, 100). Following MacCallum et al.’s (2006) recommendations, sample size was calculated using RMSEA values and pairs in the midrange of the scale (specifically, 0.03 versus 0.06). To achieve the desired power of 0.80, a sample size of 174, 200, or 273 would be required for *df* of 50, 80 or 100 respectively. This suggests that a sample size of 284 participants was powered to detect moderate differences.

Bootstrapping (2000 samples) was performed for all analyses to account for minor deviations from normality, and, in line with Valente et al.’s (2016) recommendations, bias-corrected confidence intervals were used to test for significance of mediated effects. Model fit was assessed through a relative chi-square test value between 1 (Byrne, 1989) and 3 (Kline, 2015), a Comparative Fit Index (CFI; Bentler, 1990), Incremental Fit Index (IFI) and Tucker Lewis Index (TLI; Tucker & Lewis, 1973) of >.90 indicating adequate fit (Hu & Bentler, 1999) and of >.95 indicating good fit (Hooper et al., 2008), and Root Square Error of Approximation (RMSEA; Steiger, 1990) values and confidence intervals of <.05 or <.08 indicating good or adequate fit respectively (Hu & Bentler, 1999; MacCallum et al., 1996). Direct effects were assessed by examining bias-corrected confidence intervals for standardised regression weights. Indirect effects were calculated through user-defined estimands for each pathway, and assessed by examining bias-corrected confidence intervals.

## Results

### Descriptive statistics

Study data is available online (Devenish, 2025). Means, standard deviations and nonparametric (Kendall’s tau) correlations between study variables are presented in Table 2. Moderate to strong correlations between variables were identified (Wicklin, 2023).

**Table 2.** Means, standard deviations and correlations for study variables.

Variable (*N* = 284)	Mean (*SD*)	1	2	3	4	5
1. Inattention	16.07 (7.44)					
2 Hyperactivity / impulsivity	15.27 (7.27)	.57**				
3 Rumination	19.85 (6.65)	.33**	.39**			
4 Racing thoughts	27.95 (10.42)	.46**	.52**	.46**		
5 Anxiety	14.96 (11.95)	.33**	.43**	.52**	.56**	
6 Depression	16.63 (12.90)	.34**	.39**	.63**	.54**	.67**

Note: **Correlation is significant at the 0.001 level.

Participant scores on cut-offs on the ASRS and DASS are presented in Table 3. Dunn’s pairwise tests (using the Bonferroni correction) indicated participants with a combined presentation of ADHD (based on the ASRS cut-off scores) reported significantly more rumination, racing thoughts, depression and anxiety than participants with non-symptomatic or inattentive presentations of ADHD. Participants with hyperactive/impulsive presentations reported significantly more rumination, depression and racing thoughts than participants with no ADHD symptomology. Participants with inattentive presentations reported significantly more racing thoughts than participants without ADHD symptomatology. No other differences were identified.

**Table 3.** Indicated presence of clinical levels of ADHD, depression or anxiety symptoms.

Scale and diagnostic indicator	*N* (% total sample)
*ASRS*	
Scores did not indicate symptoms consistent with an ADHD diagnosis	187 (66%)
Exceeded cut-off for symptomatic levels of inattention only	39 (14%)
Exceeded cut-off for symptomatic levels of hyperactivity/impulsivity only	19 (7%)
Exceeded cut-off for symptomatic levels of combined presentation	39 (14%)
*DASS*	
Anxiety scores within a normal range	94 (33%)
Anxiety scores within a mild range	19 (7%)
Anxiety scores within a moderate range	44 (15%)
Anxiety scores within a severe range	32 (11%)
Anxiety scores within an extremely severe range	95 (33%)
Depression scores within a normal range	102 (36%)
Depression scores within a mild range	32 (11%)
Depression scores within a moderate range	47 (16%)
Depression scores within a severe range	28 (10%)
Depression scores within an extremely severe range	75 (26%)

### Demographic characteristics in relation to study variables

Study variables did not significantly differ by gender. Age was significantly and negatively related to all study variables. Participants with higher levels of education or employment reported reduced hyperactivity/impulsivity, anxiety, and depression. Participants with higher levels of education also reported reduced inattention, racing thoughts and rumination.

The study sample size was not ample for inclusion of demographic variables in the model. To provide an indication of whether gender, education, and employment may act as confounding variables, parallel moderated mediation models between each subscale of the ASRS, and each outcome variable, were conducted via Hayes (2022) PROCESS macro. Rumination and racing thoughts were included as mediators, and gender, level of education, and employment status were included as covariates. Age and employment were not identified as significant covariates in these models. Education was identified as a significant covariate in a model that tested pathways between inattention and depression, however rumination and racing thoughts still remained significant mediators in this model.

Given that medication use could reduce symptoms of ADHD, anxiety or depression, *t*-tests were conducted, which indicated that participants who use medication experienced higher levels of ADHD, anxiety and depression symptoms. The overall study sample size, and the size of the participant groups taking medication that may impact their attention (*n* = 41) or mood (*n* = 67), were not sufficient for inclusion of medication in the model as a moderator. To account for this, parallel moderated mediation models via Hayes (2022) PROCESS macro were conducted for each subscale of the ASRS against each outcome variable. No moderation by medication was identified in any model.

#### Full Model

**Figure 3.**
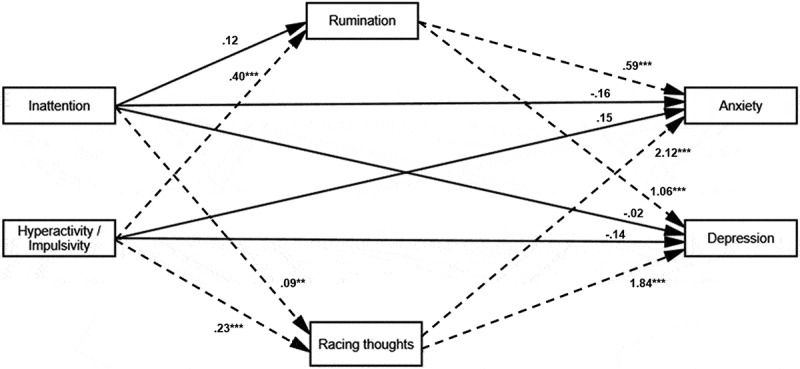
Results of the structural equation modelling analysis: Racing thoughts and rumination as mediators between inattentive and hyperactive/impulsive symptoms and depression and anxiety symptoms.

*Note*. **p < 0.01 level. ***p < 0.001 level.

To test the hypotheses that rumination and racing thoughts mediate the relationship between inattentive and hyperactivity/impulsivity characteristics with depression and anxiety scores, a structural equation model of the full model was conducted. The chi-square for independence model was significant, indicating the variables were correlated, *X*^2^ (10, *N* = 284) = 16.07, *p* = .10. Model fit was good (relative chi-square = 1.61, *df* = 10; CFI = .99; IFI = .99; TLI = .99; RMSEA = 0.05 [0.07, 0.09]). The model and results of the measurement model are presented in Figure 3 and Table 4 respectively.

**Table 4.** Standardised and unstandardised regression weights, standard errors, construct reliability and bias-corrected *p* values and 95% confidence intervals of the structural model.

Label	Path	β	B *(SE)*	*CR*	*p*	95% BCCI	
Anxiety							
*Direct Pathways*							
	Inattentive characteristics → Anxiety	−.10	−0.16 (0.09)	−1.68	.15	− .35	0.05
	Hyperactivity/Impulsivity characteristics → Anxiety	.09	0.15 (0.11)	1.37	.32	− .12	0.42
*Indirect Pathways*							
*a*	Inattentive characteristics → Rumination	.13	0.12 (.07)	1.74	.07	− .01	0.27
*b*	Rumination → Anxiety	.33	0.59 (.09)	6.50	.001	0.39	0.79
*c*	Inattentive characteristics → Racing thoughts	.21	0.09 (.03)	3.13	.003	0.03	0.14
*d*	Racing thoughts → Anxiety	.54	2.12 (.27)	7.82	.001	1.51	2.74
*f*	Hyperactivity/Impulsivity characteristics → Rumination	.44	0.40 (.07)	5.71	.002	0.25	0.54
*g*	Hyperactivity/Impulsivity characteristics → Racing thoughts	.56	0.23 (.03)	8.06	.001	0.18	0.30
*a → b*	Inattentive characteristics → Rumination → Anxiety	.04	0.07 (.04)		.07	− .00	0.17
*c → d*	Inattentive characteristics → Racing thoughts → Anxiety	.11	0.18 (.07)		.002	0.06	0.32
*f→ b*	Hyperactivity/Impulsivity characteristics → Rumination → Anxiety	.14	0.23 (.06)		.001	0.14	0.36
*g → d*	Hyperactivity/Impulsivity characteristics → Racing thoughts → Anxiety	.30	0.50 (.10)		.001	0.32	0.71
Depression							
*Direct Pathways*							
	Inattentive characteristics → Depression	−.01	−0.02 (.09)	− 0.22	.83	− .24	0.18
	Hyperactivity/Impulsivity characteristics → Depression	−.08	−0.14 (.10)	− 1.36	.23	− .37	0.08
*Indirect Pathways*							
*a*	Inattentive characteristics → Rumination	.13	0.12 (.07)	1.74	.07	− .01	0.27
*h*	Rumination → Depression	.55	1.06 (.09)	12.00	.001	0.86	1.27
*c*	Inattentive characteristics → Racing thoughts	.21	0.09 (.03)	3.13	.003	0.03	0.14
*i*	Racing thoughts → Depression	.44	1.84 (.26)	7.01	.001	1.19	2.50
*f*	Hyperactivity/Impulsivity characteristics → Rumination	.44	0.40 (.07)	5.71	.002	0.25	0.54
*g*	Hyperactivity/Impulsivity characteristics → Racing thoughts	.56	0.23 (.03)	8.06	.001	0.18	0.30
*a → h*	Inattentive characteristics → Rumination → Depression	.07	0.13 (.08)		.07	−0.01	0.29
*c → i*	Inattentive characteristics → Racing thoughts → Depression	.09	0.16 (.06)		.002	0.06	0.30
*f → k*	Hyperactivity/Impulsivity characteristics → Rumination → Depression	.24	0.42 (.08)		.001	0.27	0.59
*g → i*	Hyperactivity/Impulsivity characteristics → Racing thoughts → Depression	.24	0.43 (.10)		.001	0.27	0.65
Covariances							
	Hyperactivity/Impulsivity ↔ inattentive characteristics		40.84 (4.02)	10.15	<.001	−0.23	0.09
	Rumination ↔ Racing thoughts		5.35 (.83)	6.48	<.001	−0.07	0.02
	Depression ↔ Anxiety		24.42 (3.53)	6.91	<.001	−0.69	0.10

As seen in Table 4, the 95% CIs of the bias-corrected bootstrap results found that direct effects of inattentive characteristics on anxiety or depression scores were not significantly different from zero. The 95% BCCIs of the bootstrap results identified significant indirect effects of inattentive characteristics through racing thoughts on depression, *b* = .16, *SE* = .06, *p* = <.01, 95% BCCI: 0.06, 0.30, and anxiety, *b* = .18, *SE* = .16, *p* = <.01, 95%BCCI: 0.06, 0.32. The 95% BCCIs of the bootstrap results did not identify significant indirect effects of inattentive characteristics on depression or anxiety through rumination.

The 95% CIs of the bias-corrected bootstrap results found that the direct effect of hyperactivity/impulsivity characteristics on depression and anxiety scores was not significantly different from zero. The 95% BCCIs of the bootstrap results identified significant indirect effects of hyperactivity/impulsivity characteristics through racing thoughts on depression, *b* = 0.43, *SE* = .10, *p* = <.01, 95% BCCI: 0.27, 0.65, and anxiety, *b* = 0.50, *SE* = .10, *p* = <.01, 95% BCCI: 0.32, 0.7. The 95% BCCIs of the bootstrap results also identified significant indirect effects of hyperactivity/impulsivity characteristics through rumination on depression, *b* = 0.42, *SE* = .08, *p* = <.01, 95% BCCI: 0.27, 0.59, and anxiety, *b* = 0.23, *SE* = .06, *p* = <.01, 95% BCCI: 0.14, 0. 36.

## Discussion

This study is the first to examine whether both racing thoughts and rumination mediate the relationship between inattentive and hyperactive/impulsive symptoms of ADHD and depression and anxiety symptoms in a general population sample. All correlation and regression weight values related to hyperactivity/impulsivity symptoms were observably larger than those of inattentive symptoms, which suggests that hyperactivity/impulsivity symptoms may be more closely related to depression and anxiety symptoms, and racing thoughts and rumination, than inattentive symptoms are. In line with our predictions, the relationship between hyperactive/impulsive symptoms of ADHD and depression was mediated by racing thoughts and rumination, and the relationship between hyperactivity/impulsivity symptoms and anxiety was mediated by racing thoughts, with rumination also identified as a significant mediating pathway. In contrast to our predictions, racing thoughts mediated the relationship between inattentive symptoms of ADHD and depression and anxiety, with rumination not identified as a significant mediating pathway.

### Racing thoughts

The findings that racing thoughts were a significant pathway between hyperactivity/impulsivity symptoms and depression and anxiety symptoms support and extend on previous studies that identified an association between racing thoughts and hyperactive symptoms of ADHD (Bertschy et al., 2020; Martz et al., 2021), anxiety symptoms (Bertschy et al., 2020; Weiner et al., 2021), and depression symptoms (Bertschy et al., 2020; Piguet et al., 2010). These findings support suggestions by Martz et al. (2021) that racing thoughts may be a form of “thought hyperactivity” experienced by people with ADHD, with the current study’s findings indicating that this thought hyperactivity may be implicated in the co-occurrence of depression and anxiety symptoms in ADHD populations.

While Martz et al. (2021) found inattentive symptoms were not related to racing thoughts once hyperactivity/impulsivity and anxiety symptoms were accounted for, our study identified racing thoughts as a significant mediating pathway even when variance between these other variables were accounted for. It is possible that the use of a general population in the current study, rather than a clinical population as per Martz et al.’s (2021) study, may explain these differing findings. However, it is also possible that the larger and more diverse sample in the current study was better able to identify a significant relationship while accounting for variance between other variables. It therefore seems plausible that the current study’s findings of a significant relationship between racing thoughts and inattentive characteristics may reflect and align with Miller and Prevatt’s (2020) proposal of an inverse association of inattentive symptoms, in which increased levels of inattention may co-occur with increased racing thoughts. Given this co-occurrence of heightened inattention and racing thoughts was significantly linked to increased depression and anxiety symptoms, further exploration of the role cognitive hyperactivity and hypoactivity may play in mental health is warranted.

### Rumination

Our study findings suggest that rumination may act as a mediating pathway between hyperactive/impulsive symptoms and depression and anxiety symptoms, which aligns with previous studies focused on hyperactive/impulsive symptoms (Horibe & Hasegawa, 2020; Kandeğer et al., 2023). However, in the current study, no significant pathway was identified between inattentive symptoms and rumination, which conflicts with previous research that identified rumination as a significant mediator between inattentive symptoms of ADHD and depression symptoms (Horibe & Hasegawa, 2020).

The model used for the current study accounts for variance-related racing thoughts when evaluating the inattention-rumination-depression/anxiety pathways. All pathways involving racing thoughts were significant mediators in the model, suggesting that racing thoughts may be a particularly strong contributing factor to symptoms of inattention, hyperactivity/impulsivity, depression and anxiety. It therefore seems possible that the current study was able to account for a confounding factor missed in previous studies – specifically, cognitive hyperactivity. While at face value this may appear to conflict with the findings of studies that had suggested the opposite trend may occur – that is, that cognitive hypoactivity (akin to CDS) may account for the relationship between inattentive symptoms and rumination (Fredrick et al., 2020) – it does align with Miller and Prevatt’s (2020) theory of an inverted relationship of attention with cognitive activity. This raises the intriguing possibility that both cognitive hypoactivity and hyperactivity may occur for adults with inattentive symptoms of ADHD, and that this may be a particularly predictive factor for depression and anxiety symptoms for adults with ADHD.

For adults with inattentive symptoms of ADHD, the findings suggest it is possible that rumination may be less predictive of depression and anxiety symptoms than racing thoughts. This potentially conflicts with previous theories (e.g., Beevers, 2005; Eysenck et al., 2007), in that our findings suggest that difficulties regulating the pace of thinking may cause more psychological distress than interrupting ruminative thoughts. Conversely, it is also possible that difficulties regulating the pace of thinking may be directly associated with, or worsen, difficulties with redirecting attention away from negative thoughts. In comparison, given that rumination remained a significant and equivalent predictor of depression and anxiety symptoms for adults with hyperactive/impulsive symptoms of ADHD, it appears that both perseverative *and* racing thoughts may hold similar predictive value for this cluster of symptoms. Since structural equation modelling by Horibe and Hasegawa (2020) found that hyperactive/impulsive symptoms, but not inattentive symptoms, were indirectly associated with rumination (and thereby depression) via interpersonal conflict, it is possible that increased externalising behaviours and interpersonal difficulties experienced by adults with hyperactive/impulsive presentations may result in increased rumination that is sustained independently of the pace of thoughts. Similarly, adults with hyperactive/impulsive symptoms tend to experience higher levels of emotional lability (Martz, Weiner, & Weibel, 2023), and it therefore seems highly plausible that this heightened emotional liability may increase a propensity to ruminate beyond the effects of racing thoughts.

### Limitations and future directions

A number of limitations exist within the current study. First, the use of a general population may limit generalisability to a clinical population, and the participants in the current study appeared to have higher scores on the ASRS and DASS than those identified in non-clinical samples by Adler et al. (2019). This may indicate an opt-in bias from participants experiencing elevated symptoms of ADHD, depression and anxiety. The study used a convenience sampling approach across two different platforms. Further, the study included participants from diverse cultural backgrounds, and did not require participants to disclose if they had received a formal diagnosis for ADHD, depression, anxiety or other medical or psychological conditions, with medication use related to attention and mood indicating there was variability in the sample. While some analyses of demographic variables occurred, the sample size did not allow for inclusion of demographic variables in the model, which may limit the generalisability of findings.

The current study was cross-sectional, and so we cannot infer whether racing thoughts simply coincide with depression, anxiety and ADHD symptoms, or are a contributing factor to these conditions. For example, while it is possible that racing thoughts are a by-product of ADHD symptoms, which may then increase depression and anxiety, it seems likely that multiple interactions between these symptoms occur, such as physiological symptoms of anxiety resulting in increased racing thoughts and reduced attention regulation. Further, it seems likely that negative external experiences may also contribute to this relationship.

While cognitive hypoactivity (akin to CDS) was not measured in the current study, limiting our ability to draw conclusions, our findings do support the possibility that deviations in an individual’s pace of thinking may hold explanatory value for understanding the relationship of ADHD symptoms with depression and anxiety symptoms, and in particular, that an acceleration in thinking may be of particular interest. The experience of racing thoughts by people with ADHD may be distressing in and of itself (Keizer et al., 2013), which could suggest that racing thoughts may themselves increase depression and anxiety. Conversely, it is also possible that racing thoughts and cognitive hypoactivity could interfere with an individual’s capacity to process and regulate internal thinking, diminishing their ability to use reappraisal (Riepenhausen et al., 2022) and mindfulness (Conversano et al., 2020) as emotion regulation strategies. This would have clear implications for treatment of depression and anxiety symptoms in adults with higher levels of inattention or hyperactivity/impulsivity. For example, mindfulness and acceptance-based approaches, which have been proposed as a potentially effective intervention for addressing racing thoughts in adults experiencing insomnia (Weiner et al., 2021), may also have increased effectiveness for addressing symptoms in adults with ADHD symptoms. Given the scarcity of literature, further research is needed to establish the relationships, including directionality and interactions, between cognitive hyperarousal or hypoarousal, ADHD symptoms, depression and anxiety symptoms, and potential treatment approaches.

## Conclusion

This study was the first to examine whether both racing thoughts and rumination are pathways linking symptoms of ADHD with depression and anxiety symptoms. Our findings suggest that hyperactivity/impulsivity symptoms may be more closely related with depression and anxiety symptoms in the general population than inattentive symptoms and provide initial evidence that racing thoughts is a key process accounting for the relationship between symptoms of ADHD and depression and anxiety symptoms. Further, rumination may also significantly contribute to the relationship between hyperactive/impulsive symptoms and depression and anxiety. Future research is needed to replicate these findings in clinical populations, and to examine whether an inverse relationship between inattention and cognitive hyperactivity and hypoactivity may be implicated in ADHD, depression, and anxiety disorders.

## Data Availability

Study data is available online: Devenish, B. D. (2025). The Role of Rumination and Racing Thoughts in Co-Occurrence of ADHD Symptoms, Depression and Anxiety Symptoms (Version 1) [Data set]. Mendeley Data. https://doi.org/10.17632/cwrtd5tbd8.1
